# Measuring Smoothness as a Factor for Efficient and Socially Accepted Robot Motion

**DOI:** 10.3390/s20236822

**Published:** 2020-11-29

**Authors:** Silvia Guillén Ruiz, Luis V. Calderita, Alejandro Hidalgo-Paniagua, Juan P. Bandera Rubio

**Affiliations:** 1 Department of Electronic Technology, University of Málaga, 29071 Málaga, Spain; silguirui@uma.es (S.G.R.); ahidalgo@uma.es (A.H.-P.); jpbandera@uma.es (J.P.B.R.); 2 Department of Mechanic Engineering, Computer, and Aerospace Science, University of León, 24007 León, Spain

**Keywords:** smoothness, path planning, energy consumption, social navigation, multi-objective optimization, robotics

## Abstract

Social robots, designed to interact and assist people in social daily life scenarios, require adequate path planning algorithms to navigate autonomously through these environments. These algorithms have not only to find feasible paths but also to consider other requirements, such as optimizing energy consumption or making the robot behave in a socially accepted way. Path planning can be tuned according to a set of factors, being the most common path length, safety, and smoothness. This last factor may have a strong relation with energy consumption and social acceptability of produced motion, but this possible relation has never been deeply studied. The current paper focuses on performing a double analysis through two experiments. One of them analyzes energy consumption in a real robot for trajectories that use different smoothness factors. The other analyzes social acceptance for different smoothness factors by presenting different simulated situations to different people and collecting their impressions. The results of these experiments show that, in general terms, smoother paths decrease energy consumption and increase acceptability, as far as other key factors, such as distance to people, are fulfilled.

## 1. Introduction

Mobile robots are gaining growing importance in the current society. In the last years, these robots are becoming more present, not only in controlled or specific environments but in daily life scenarios. When they are designed following a correct user-centered process that focuses on efficiently using their abilities, robots achieve very successful results. These results make them a promising technology that has the potential to change the society habits [1]. Hence, mobile robots are increasingly being used in different application areas, such as rescue tasks, exploration, supervision, warehouse transportation, goods delivery, or people assistance. The tasks which these robots address in these application areas are also evolving, becoming more complex and diverse. For instance, autonomous drones can not only be teleoperated but automatically collect a lot of information about natural disasters, allowing fast responses to help involved people. UAV (unmanned aerial vehicles) and AGV (autonomous ground vehicles) are currently used not only in dangerous tasks (nuclear disasters, bomb disposal, etc.) but in more common scenarios, such as delivery tasks or goods transport. Moreover, robots are beginning to be used in scenarios in which they cooperate with or assist people, interacting through natural and intuitive channels. Assistant robots can be used to monitor elderly people in retirement houses, acting as social facilitators, helping caregivers in their daily life activities, and even detecting risky situations and triggering alarms [2]; moreover, they can be used to teach therapeutic exercises to patients [3] or drive geriatric assessment procedures [4].

In order to get all these functionalities, mobile robots require an adequate navigation system. In daily life environments, navigation requires the robot to calculate how to move around people following not only safety criteria but social conventions, while reaching a goal point. These calculations are obtained using Path Planning (PP) algorithms. There are a lot of different algorithms that can be used and combined to achieve feasible path planning (e.g., those based on Evolutionary Computing (EC) or Artificial Intelligence (AI) techniques, among others); the election depends on what factors are relevant for the robot. Among these factors, three are usually considered to evaluate the trajectory of a robot: path length, path safety, and path smoothness [5,6].

The importance of these factors should be prioritized taking into account the purpose to be fulfilled by the robot and its characteristics. In the case of mobile robots, one common and imposing constraint is energy consumption: robots should save as much energy as possible to become more useful devices and be able to perform not only more tasks but more complex tasks in the same amount of time. A robot able to work without recharging for a long time undoubtedly becomes a better asset in a person caring, rescue tasks, or autonomous goods transportation.

While the path lengthis usually considered the most significant factor in battery consumption, there are situations where the shortest path has closed angles. These turns can imply a higher energy consumption (except for omni-wheels robots). This is mainly because the robot will continuously change the rotation direction of its motors. Moreover, these types of paths can be perceived as discontinuous and odd, especially for a social context [7].

On multi-objective path planning, it is common to use a fitness function that associates each objective with a number which symbolizes its importance in the final path [5,8,9]. Usually, path safety referred to an obstacle-free path, has the highest importance, followed by the path length and, finally, the path smoothness. However, even if smoothness may not be the main factor related to energy consumption, its influence cannot be neglected in any scenario. Moreover, when considering robots that share their environment with people, smoothness acquires a new dimension, related to produce socially accepted paths [10].

In addition, giving maximum importance to path length can also lead to situations of increased energy consumption, for instance, bordering a square table by making an abrupt path that moves forward, stops, and turns 90 degrees. This behavior is not only inefficient, but it also does not seem to be the most appropriate from the point of view of our social conventions.

The objective of this paper was to measure the importance of the smoothness factor, both to (i) achieve socially accepted trajectories for robots working in daily life scenarios; and (ii) produce more efficient trajectories, increasing the autonomy of the robot. How these two dimensions of path planning correlate—or not—through the smoothness factor, is one of the main contributions this paper pursued.

The experiments performed to achieve these objectives involve measuring the energy consumption on fourteen different paths. These paths have the same starting and ending points but different smoothness degrees. The robot is going to walk through each of them with the same velocity, while energy consumption is measured. These measures will be analyzed later in order to determine the relation between energy consumption and the smoothness factor.

Social acceptance, on the other hand, is going to be analyzed through surveys. These surveys, filled by different people who observe the robot while it performs the different paths, it collects information, and it detects what behaviors are perceived as more comfortable and friendly. As in the experiments related to energy consumption, different trajectories with different smoothness factors are employed to analyze the effects of this factor in the acceptance of the robot’s motion.

The rest of the paper is organized as follows: In Section 2, a state-of-the-art in path planning algorithms is provided. Then, Section 3 describes the smoothness factor, how it is calculated, and its implications on path planning. Section 4 describes the simulations that have been created. Smoothness will be evaluated on two different factors: how it influences on energy consumption (Section 5) and social acceptability (Section 6). Section 7 discusses the correlations between energy efficiency and acceptability, regarding variations in the smoothness factor. Finally, Section 8 summarizes this work and remarks on the main obtained results and future research lines.

## 2. Related Work

Many algorithms have been proposed in recent years to perform path planning. These proposals try to find feasible paths, taking into account different factors, that depend on the characteristics and functionality of the used robot. For instance, in Reference [11] a path planning algorithm based on clothoid curves is employed to produce continuous curve paths. They focus on smoothness and obtained better results than their predecessors. In fact, the smoothnessfactor is a priority when smooth and continuously trajectories are required, for instance, when programming the navigation systems for autonomous vehicles. Reference [7,12] propose an evolution of the aforementioned work, in which both approaches use polynomial path planning. While the latter ignored vehicle sideslip due to cornering velocity, in general, these two studies obtained feasible, safe, and smooth trajectories.

Most path planning algorithms do not focus only on maximizing a single factor, such as the previously mentioned works devoted to the path smoothness. Typically, these algorithms address also minimizing the path length, and maximizing the path safety. Hence, Reference [9,13] propose the use of the elitist NSGA-II (non-dominated sorting genetic algorithm) [14] to simultaneously compute different solutions to PP. To obtain these paths, a set of factors in a fitness function are modified, and these modifications produce paths with different features. The final paths are compared and the better ones are selected, setting the fitness function to be used. Furthermore, authors propose as future works, adding more factors to the fitness function, including energy consumption.

Other algorithms that consider path length and safety, as in Reference [15,16,17], also introduce the concept of cost-map. The area where the robot is going to move is represented as a grid divided by cells. These cells could be free, fully occupied by an obstacle, or partially free. Then, the cost-map has a different output depending on the occupancy, ranging from a minimum cost for free cells to infinite cost for fully occupied cells. This cost-map representation is used to compute a path using D*-PO and A*-PO algorithms. In these studies, these algorithms can solve and optimize path planning, achieving better computation times than traditional A* and D* algorithms.

A variation of A* algorithm is presented in Reference [18], with path segments planned using FMM and PSM. This algorithm divides the path planning procedure into two independent phases: path planning and path assembling. It has a good computation time but the obtained path length is longer than the optimal one. A variation using heuristic on A* and D* algorithms is implemented in Reference [19]. They obtain good path planning results, reducing the computation cost, although this cost depends on the number of matrices to calculate. On this line of algorithms appears Reference [20]; in this proposal, the factor path safety is maximized and computation cost is minimized using an optimized Dijkstra algorithm to find a path between a source and goal node on a grid map.

Ahmed and Deb [5] propose a modification of NSGA-II that adds another factor: path smoothness. Path length and path vulnerability are used as two objectives on the path planning process, in which, as in the other works, space is represented as a grid. The number of interfering cells is used as a constraint and is handled using the penalty function approach. The smoothness factor is included when the algorithm compares solutions having the same rank. The solution with the best smoothness value is chosen. Besides, the smoothness value is also used on the post-processing stage when two solutions coincide at the same point. The best solution is the one with the largest smoothness value, where open angles are considered smoother than narrow angles.

In these contributions, smoothness is presented as a factor to take into account because it helps to obtain a continuous path that reduces slippages. Fewer slippages lead to less energy consumption and less travel time. Simulation results demonstrate that this variation allows finding feasible paths more accurately, even on large-size environments with a high density of obstacles (up to 91% of space). The inherent diversity preserving the ability of multi-objective genetic algorithm (GA) allows finding solutions in such difficult scenarios, in which conventional techniques or single-objective genetic algorithms may find serious difficulties finding an adequate path.

Biologically inspired approaches take this idea of using three objectives to find feasible paths a step further. In Reference [6], Frog-Leaping algorithms are used, and, in Reference [21], fireflies behavior based algorithms are also used to solve path planning. In Reference [8], a MOVNS (Multi-Objective Variable Neighborhood Search) is used for the first time to solve the path planning for mobile robots; in this contribution, the smoothness factor is not simply used as a discriminant for coincident situations, but as another target on its own. Therefore, in Reference [8], the fitness function is modified to obtain a better relation between the three factors. The best combination is selected depending on what combination gets the best path planning solutions. These three contributions obtain better path planning solutions than classic NSGA-II. They also discuss a relation between smoothness and energy consumption.

Another example of multi-objective path planners that consider these three factors (path length, path safety, and path smoothness) is found in Reference [22]. Nevertheless, in this case, smoothness is treated as a secondary objective. The variation on this algorithm consists of developing a novel hierarchical global path planning algorithm in a cluttered environment, minimizing the path length, and maximizing the path smoothness with a particle swarm optimization with an accelerated update methodology based on Pareto, getting an efficient computational time algorithm. To sum up, this method has three steps of computation: first, the obstacles, space limitations, and space of safety are set, and the triangular decomposition method is applied to find free space; the second step uses Dijkstra’s algorithm to generate the collision-free path; finally, Multi-Objective Particle Swarm Optimization (MOPSO) function is employed to obtain the optimal path, based firstly on path length and then on path smoothness. Some proposed methods are able to avoid prioritizing one factors over others, while still obtaining efficient results. Hence, Reference [23] or [24] describe path planners that are able to compute the shortest paths, while considering smoothness criteria, and they do so without increasing the computation times.

Table 1 summarizes the main characteristics of all the different references about path planning methods evaluated in this paper. The main factors that influence path planning are smoothness, path length, and path safety. Social acceptance has only been employed as a factor in one of the papers. As depicted, most of the multi-objective path planning algorithm employs fitness functions.

In order to compare these approaches, they can be divided into two big groups: uni-objective path planners and multi-objective path planners.

Uni-objective path planners are useful only for specific purposes, where it is possible to always prioritize a certain factor above all.

Multi-objective path planners, on the other hand, allow prioritizing the factors which are considered more relevant for each situation. Furthermore, their capability of involving more factors makes them more adequate to adapt to different contexts and to react in dynamic unpredictable settings, such as daily life scenarios. The use of fitness function, in particular, adds the advantage to adapt the path planning to each particular situation just adjusting the factors in the function. This capability also eases using the same algorithms in different robots. The main disadvantage of multi-objective path planners is that they may require more computation time, depending on the considered factors. This disadvantage is not really significant, as there are already interesting path planning options [23,24] allowing the use of path length, path safety, and smoothness factors without increasing computational time.

Not all multi-objective path planners rely on the same factors. Hence, some algorithms use the three factors (maximize smoothness, minimize path length, and maximize path safety). These path planners suit contexts in which none of these factors should be neglected. Their drawback is that those resulting paths may not be the shortest ones.

The most common multi-objective path planners use two factors. Path length and path safety are usually employed, as these planners obtain the fastest paths to arrive safely at the goal. However, generated trajectories may not be efficient, nor socially adequate. Some other planners consider path safety or smoothness. While these path planners are useful for social robots, the path obtained may not be efficient in terms of consumption or path length.

## 3. Computing Smoothness Factor in Path Planning

As other authors state [6,8,21], path smoothness is a concept directly related to energy and time consumption. So, in the context of the Path Planning (PP) problem, the smoothness objective tries to minimize the number of sudden turns along the path. By sudden turns, it is referred to those turning points along the path where the robot needs to stop, turn, and start moving again.

Hence, a smooth path is one that will most likely require the robot never to stop. At most, the robot will have to reduce its speed depending on the curve. This definition leads to the intuition that a robot will take less time to traverse a smooth path than a non-smooth one. Moreover, robots should consume less energy when following smooth paths, as stop-and-start sequences for motors are avoided. These sequences represent significant consumption peaks. One objective of this paper is to check the validity of this intuition.

Following the proposals of previously referred works [21], it will use Equation (1) to compute the path smoothness.

(1) $$Smoothness=\sum_{i=1}^{n-1}min\left(\alpha \left(Sgi,Sg_{i+1}\right),\beta \left(Sgi,Sg_{i+1}\right)\right).$$

In Equation (1), *n* refers to the number of path segments. For trajectories conformed by straight segments, $\alpha (Sgi,Sg_{i+1})$ and $\beta (Sgi,Sg_{i+1})$ refer to the *angles* between the segments *i* and *i* + 1 of the path.

When analyzing curve trajectories, on the other hand, $\alpha (Sgi,Sg_{i+1})$ and $\beta (Sgi,Sg_{i+1})$ refer to the curve’s radius between the segments *i* and *i* + 1 of the path.

The objective of the path smoothness operator is to maximize the value of Equation (1). Trajectories composed by straight segments will reach their best values at 180*(*n* − 1) or π*(*n* − 1), depending on the measurement units (degrees or radians, respectively). When dealing with curve trajectories, it can not use degrees to calculate the smoothness because the robot is not sequentially performing rotations and straight motions, but it describes a continuous curve. Hence, the correct measure to compare smoothness in these cases is the radius of the followed curves. The curved paths’ objective can be directly described as maximizing the radius, where a greater radius implies a greater smoothness.

All the trajectories have been manually generated. The smoothness of trajectories composed of straight segments is easily computed by considering the number of segments and the angles between them. Curve trajectories are composed of curve segments. For each of these segments, the radius is computed. After taking the measurements, the calculations were made by entering the data manually in an Excel file. This procedure is repeated for all trajectories.

## 4. Implementation

This section describes the setup for the experiments detailed in this paper. Two well-differentiated experiments have been conducted: one analyzes the influence of the smoothness factor on energy consumption, while the other considers this influence but on the social acceptability of performed paths. As detailed above, this paper aimed to explore correlations between the two experiments towards maximizing these two factors.

### 4.1. Measuring Energy Consumption in a Real Robot

One of the objectives of this paper was to verify if there is a relation between smoothness and the energy consumption of the robot. For this purpose, it is necessary to measure the consumption of the battery on different paths, with the same length (67.2 m), and performed by the robot at the same velocity (0.25 m/s).

**Figure 1:**
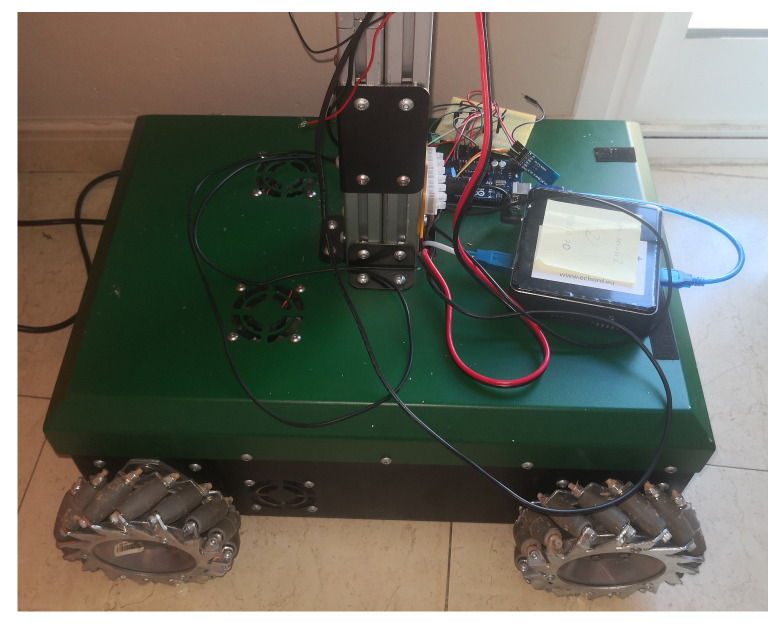
Robotic base employed in the energy consumption experiments.

In order to perform these tests, a four-wheel mecanum mobile robot base is employed (Figure 1). This robot is equipped with an embedded PC (a Intel Next Unit of Computing (NUC) with an i3-7100U processor and 4 GB of DDR4 memory RAM, running Ubuntu 18.04 and an Arduino-based system.

The NUC is connected to the platform, and it is in charge of sending the motion commands required to follow a given path. These motion commands come from an Message Queuing Telemetry Transport (MQTT) Application able to load the paths as files. These files describe each path as a set of orders, and the NUC PC converts these orders onto wheels motions. The paths which have been described on these files have the same length but a different shape (saw-shaped, curved, squared, straight).

In order to ease robot control, the MQTT application is run on a different PC that sends the motion orders to the NUC via WiFi.

**Figure 2:**
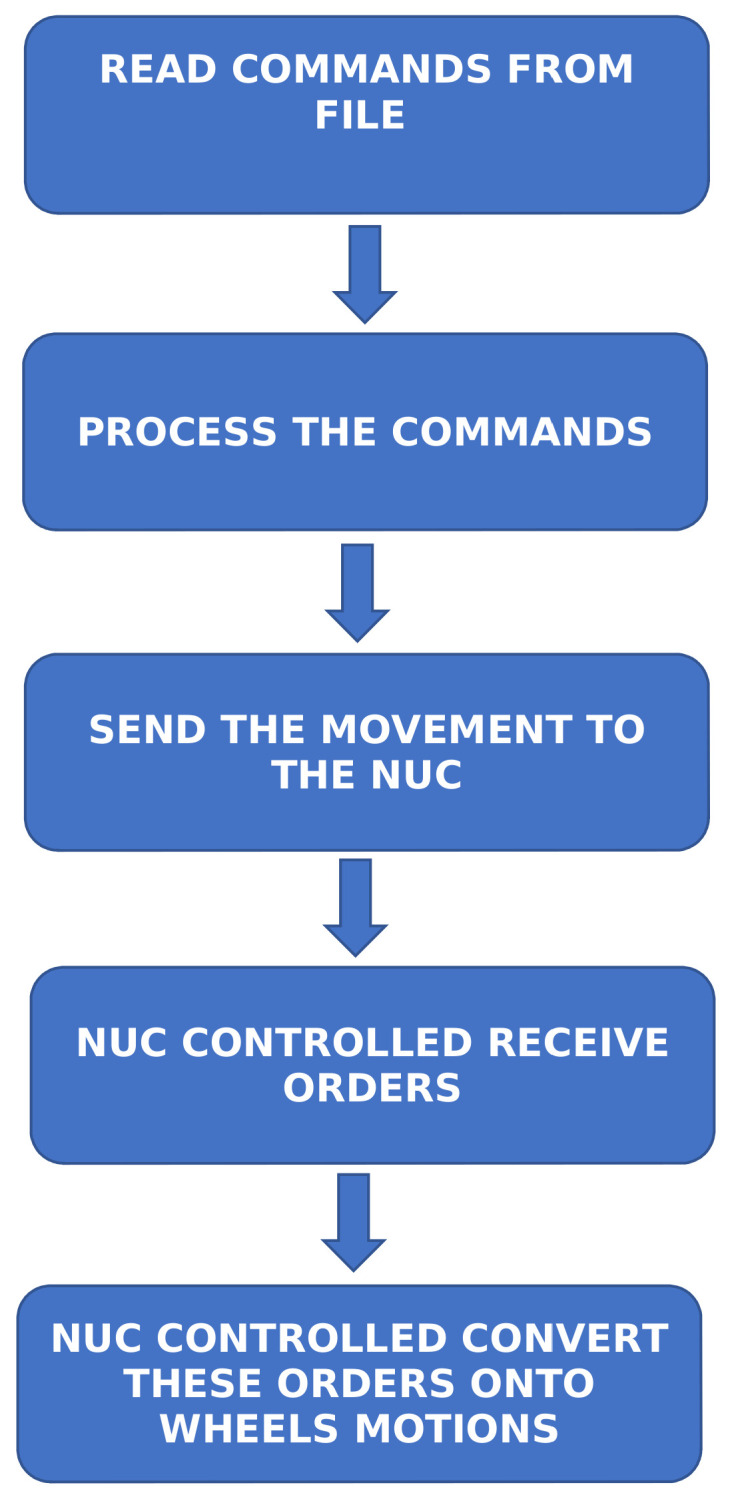
Flow diagram of the applications.

Figure 2 shows the flow diagram of the described process.

**Figure 3:**
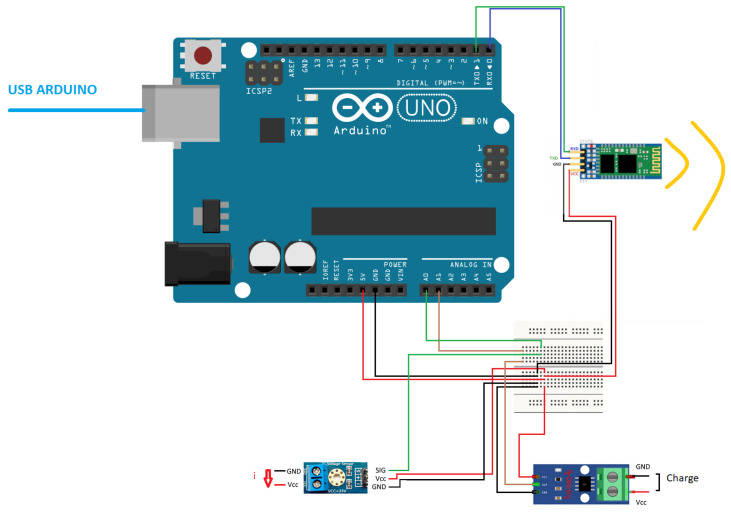
Arduino diagram.

**Figure 4:**
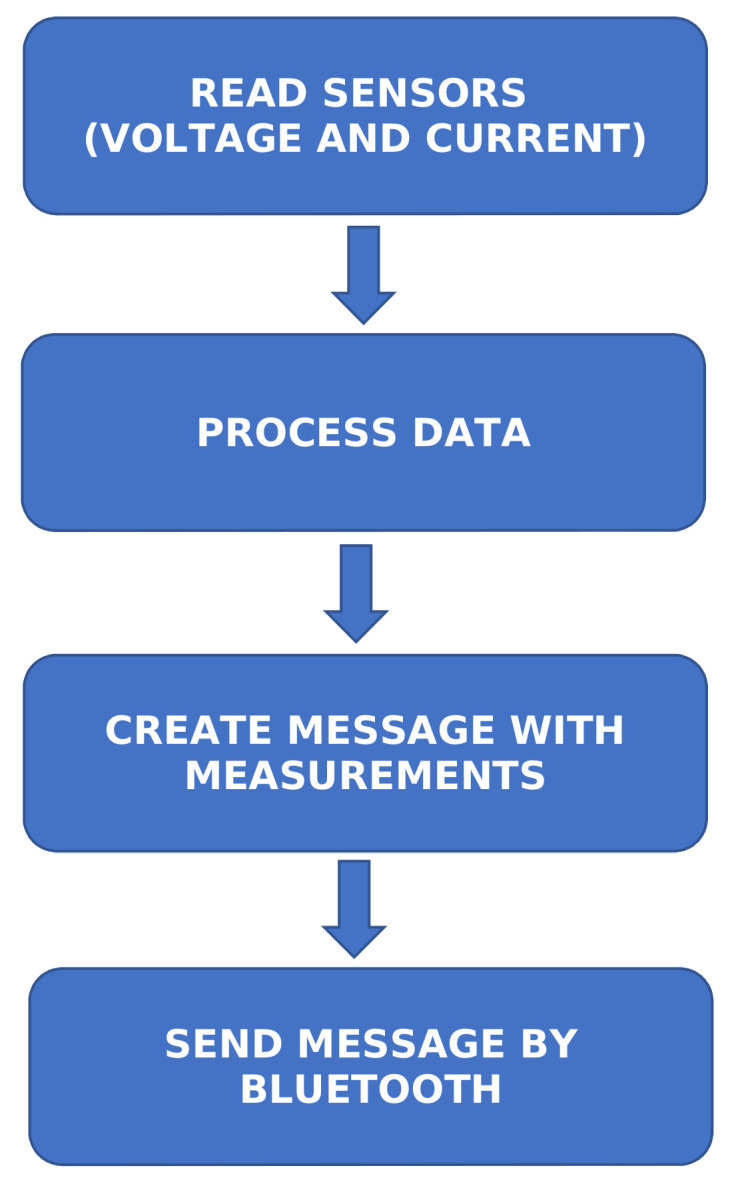
Arduino code flow.

Regarding energy measurements, the robotic base has voltage outputs that will be used to perform these measures using the system that Figure 3 depicts. It is needed to connect these output to a system which lets to measure this output on each path. The system to measure that is based on an Arduino microcontroller, an ACS712 current sensor, a FZ0430 voltage sensor, and an HC-05 Bluetooth module. The current sensor is connected in series with the main circuit and the voltage sensor in parallel with the battery charge output. Figure 4 shows the main steps of the program in charge of reading the data and sending it.

**Figure 5:**
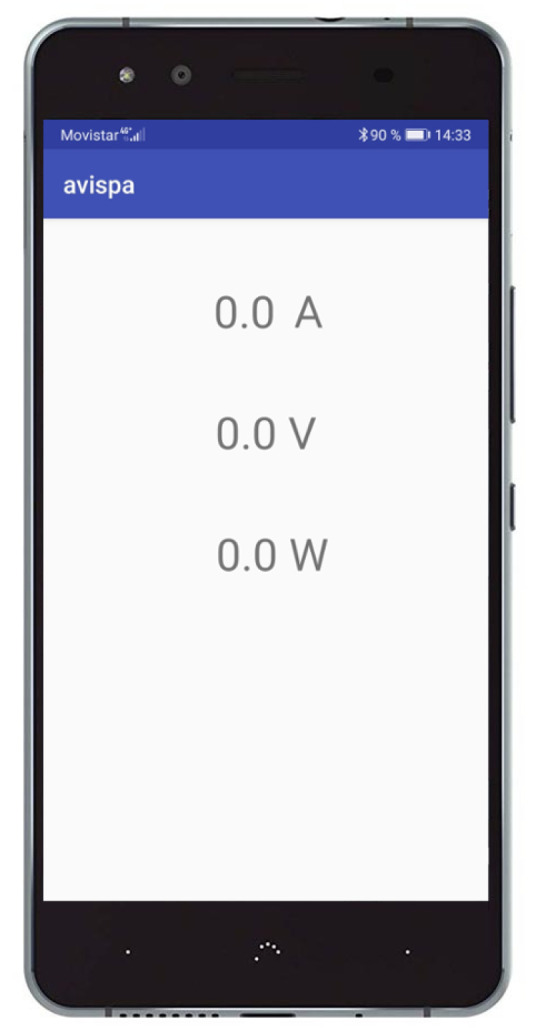
Avispa app.

However, it is needed to keep and read these measurements. To get there, it has deployed an android application named Avispa. Avispa receives via Bluetooth the measurements from Arduino and shows this on our device (Figure 5).

To keep this data, the information is also sent from Avispa to a Server. Finally, to access this data and take the measurements, an MQTT application is implemented. This application runs on the control PC, is subscribed to the server, and keeps the data on an Excel file on that PC.

### 4.2. Creating Simulations for Social Acceptance Analysis

On the other hand, it is needed to demonstrate by surveys if there is a relation between social acceptance and smoothness.

In this experiment, a group of people evaluates different simulate scenes in which a robot has to describe different paths. These scenes are created using the robotic simulator CoppeliaSim [25]. This tool is a 3D simulator that integrates a development environment. It is a multiplatform tool that allows using several programming methods, such as Robot Operating System (ROS), with a client Application Programming Interface (API) on C++, an embedded scripting language. In Windows, it could even use Visual Studio. In addition, it supports several programming languages: C++, Lula, MATLAB, Python, etc. It includes a physics engine, collisions, camera, and Kinect simulation.

Each of these use cases consists of a scenario in which a robot describes different trajectories around a certain environment. This environment is populated in each situation with different objects. In some use cases, people are present in the environment. For each scenario, the robot goes from a starting point to an ending point following different trajectories (i.e., using different smoothness factors).

To evaluate the cases, surveys were created using Google Forms. The set of use cases will be presented to a wide group of volunteers, via an online questionnaire The surveys have this structure:

- First, a set of questions related to demographic.
- On each survey, the same scene is shown, but the robots will follow different trajectories.
- To show trajectories have been recorded, videos on CoppeliaSim simulate the scene and the trajectory of the robot.
- A section for each video where the participant watches the video of the trajectory and answers a set of questions.
- A set of questions aiming to infer if the behaviors of the robot have been perceived as friendly, comfortable, safe, and socially adequate.
- Finally, two open questions ask what trajectory they prefer and why.

Google Forms save this data and collect the results on an Excel file. The Excel file is used to process the data and analyze it.

## 5. Evaluation: Smoothness Influence in Energy Consumption

The robot plus measuring system presented in Section 4.1 will follow different paths to analyze and measure the influence of the smoothness factor in energy consumption. These paths are classified into four groups: saw-shaped path, curved path, squared path, and straight path (Table 2).

### 5.1. Consumption’s Results

Table 3 and Table 4 show energy consumption for trajectories based on straight lines and curved lines, respectively. Each file in the table describes a certain test, showing a draft of the followed path, a description of the test, the movements described on the file which is loaded for that path in the MQTT application, the energy consumption measured by the Arduino system, and the average smoothness value (in these testing trajectories, all turns are equal in each test, so the smoothness standard deviation is always 0).

The first obvious conclusion these tables show is that energy consumption varies depending on the type of motion. For saw-shaped paths, lower turning angles (i.e., smoother trajectories) significantly reduce energy consumption. Hence, the squared path becomes the less efficient behavior, while turns of 15 degrees or lower are close, in terms of energy consumption, to the ideal situation (straight line), at least for these tests.

Curved paths, on the other hand, show a decreasing value of energy consumption as the curve becomes smoother. A peculiar result is that the smoothest curve paths have an energy consumption value even below the straight-line path. This effect is most probably a consequence of using four omni-wheels to move the base. The effort the motors require to keep these wheels rolling to produce a straight motion may be higher than the one required to follow a smooth curve.

In conclusion, results shown in Table 3 and Table 4 are completely coherent with the hypothesis that increasing the smoothness of the trajectories significantly decreases energy consumption.

## 6. Evaluation: Smoothness Influence in Social Acceptability

Table 5 describes the five use cases (*scenarios*) that have been prepared in the CoppeliaSim simulator. In each of these scenarios, the robot moves from a starting point to an ending point avoiding obstacles and following different trajectories. Therefore, for each scenario, a set of different situations is produced, and the robot follows a different trajectory in each situation.

As Table 5 shows, two people facing each other are included in scenarios 1 and 2. In scenario 1, the robot will move between these two people, while in scenario 2 it will surround them. Scenarios 3, 4, and 5 are only populated with objects.

*Situation* i for *scenario* X is labeled as X.i. Table 6 shows the characteristics of the motion performed by the robot in each of these situations. The videos showing each motion can be checked in the supplementary material.

In these scenarios, the robot will follow trajectories in which not all turns will be equal. Hence, the smoothness of the trajectory cannot be defined by a single value, like in Section 5.1. It has been provided instead of an average smoothness value, along with a standard deviation value. In the simulated environments employed for these tests, the coordinates of each discrete simulation sample are used as segment limits. Table 7 shows obtained smoothness, where, in general terms, X.1 situations are smoother than X.2 when dealing with straight segments, and X.4 curves are smoother than X.3 ones.

### 6.1. Questionnaire

The survey starts with a section that collects demographic data: gender, age, and use of technology. Then, for each situation, the participants have to answer a set of questions using a Likert scale. Table 8 links refer to a copy from the original in order not to modify given responses. Table 9 lists the questions.

The closing section of the survey includes two open questions (Table 10).

### 6.2. Survey’s Results

The survey was distributed online using different channels (e-mail, news channels, distribution lists of University Departments and Faculties, etc.). It was split into four episodes (one per scenario, except the fourth fragment that included scenarios 4 and 5), to avoid a single time-consuming activity. This decision had also related issues: not all people starting the survey completed it. In fact, the first scenario is evaluated from the answers of 46 people, the second one counted with 24 responses, the third scenario 21, the fourth 15, and the fifth 14 (one person answered only one part of the last fragment of the questionnaire). Table 11 summarizes the demographic data of the people who participated in the questionnaire (answering at least the questions about the first scenario). Table 12 exposes the experience of the participants about using technology.

Table 13 shows the mean values and standard deviations for the questions that are punctuated using a Likert Scale in the questionnaire (Q1–Q7). Table 14 shows the percentage of agreement for the questions that admit only Yes/No responses (Q8, Q9). All people answering each scenario have been taken into account to compute these results.

The analysis of these results offers some interesting data. First of all, in general terms, saw-shaped paths (X.2 situations) make people be confused about what the robot is doing (low scores in *Q2: The robot’s actions are clear to people*). Moreover, these saw-shaped trajectories are perceived as not socially appropriate and odd when there are people around (Q3 and Q4, situations 1 and 2). These trajectories may also distract people more easily (Q9), a logical result as people will tend to focus on the robot if they cannot understand what it is doing nor to predict its behavior.

Focusing on *Q1: The robot takes people into account*, it can be seen that surrounding people (2.x situations) are preferred over going through them (1.x situations). This result was expected, but not the good score obtained for squared surrounding trajectories (Q1, 2.1). The free comments of the participants in the survey help to understand this result: this 2.1 trajectory makes the robot keep more distance from people. Moreover, it transmits tranquility by not having many changes of direction.

Questions Q2 and Q7 are the main items to be considered when analyzing if people can understand the actions of the robot. The results show that the smoothest paths (X.4) are the ones more easily anticipated. Again, this is a logical conclusion since once the observer realizes that the robot moves smoothly, she will not expect it to make abrupt turns, and will be able to predict its short-term destination more easily.

The robot going directly to the goal (situation 1.0) is a special case for this analysis. This trajectory is well understood and predicted, even if it is not respecting social conventions at all: observers understand that the robot, needing to go to the table, can annoy a little bit the people by going between them because it is something that most people would do (as the high score of situation 1.0 in question Q4 reflects).

Regarding how social the navigation of the robot is perceived (questions Q3 and Q5), apart from the bad results for saw-shaped trajectories previously mentioned, the results show that surrounding people with smooth curves is the more adequate behavior (much better than passing between them). If no people are present, in general terms curve trajectories are better perceived, especially if they make the robot keep more distance from people. It is also interesting to highlight that a path that is significantly shorter than other alternatives (such as 1.0) will be associated with *what a person would do* (question Q4). However, when performed by a robot, these direct trajectories, which may be breaking social conventions (situation 1.0) or moving too close to objects (situation 4.2), can create a sensation of threat or annoy people (results of questions Q6 and Q8). So, it could be concluded that, to be accepted by people, the robot may not focus on behaving like a person but on keeping distance, performing smooth motions, and following social rules *more strictly than people themselves*.

Questions Q6 and Q8 focuses on the robot being perceived as a threatening or annoying presence for the people. These results are quite related to previous questions Q3 and Q5, and they show that very smooth motions, which keep the robot away from objects and people, are perceived as the safest and less annoying options. In fact, while paths following very smooth curves convey a feeling of tranquility and familiarity, this same feeling is often produced by trajectories performing 90° turns because these trajectories tend to keep the robot away from objects and people. On the other hand, a robot that goes between people, or one that performs abrupt changes in its motion, can easily annoy and scare people, as expected. Results of question Q9 reinforce this conclusion.

Regarding the open questions at the end of the survey, it offers additional interesting insights complementing and extending the ones collected using the questionnaires for each situation.

The first open question (*OQ1: Would you have preferred some other movement of the robot?*) collected several interesting answers. Some participants suggested the use of a combination of angles and curves in the trajectory makes the robot behave more naturally. Moreover, some of them point towards the necessity of providing the robot with a higher social awareness level. For instance:

- “*The robot always crosses between the two people who are face to face and could interfere with what they are doing. Maybe I should avoid crossing in the middle and surrounding both people*”.
- “*That it (the robot) should have stopped without reaching the goal since he had to pass between two people. At least, the robot should have stopped before crossing between them and then, if necessary, go slower*”.
- “*A person would never have gotten so close to other people if it can be avoided… Why not surround them from further away? This way you avoid feeling threatened*”.
- “*I would have liked the robot to demonstrate more its presence, especially in the last video (of situation 2)*”.
- “*That it (the robot) were further away from the person*”.
- “*Further from the person or by the shortest path*”.

These suggestions match the conclusions obtained from the questionnaires used in this study, aligning with the ones reached in Reference [10]: a social robot needs to be aware not only about the obstacles and people around but also about the social context and acts according to it.

Finally, the last open question (*OQ2: Which of the 5 trajectories did you find more natural and less invasive? Justify your answer*) also reinforces previous conclusions. Most participants choose the trajectories which follow smooth curves. Some of them also point out that straight trajectories are also a natural option (as discussed above for Q4 results in situation 1.0). Some relevant answers are listed below:

- “*The line is clearly the one that seems the most natural, and among those that have to rotate, I would first put the one that makes the angles smoother*”.
- “*Making very smooth curves, it gives a feeling of greater tranquility*”.
- “*The one with very smooth curves. Smooth movement makes the robot less invasive and predictable*”.
- “*The one with smooth curves because it is very predictable*”.

The following answer is also relevant, as it highlights the importance, for a social robot, to be perceived exactly as it is, avoiding false expectations and confusion: *"The one with 90° angles. It seems less invasive to me. It also seems more predictable to me, so it increases the feeling of being less invasive. Perhaps it is an unnatural path for a person, but I see it "natural" for a robot that pretends to be less invasive in the room"*

## 7. Discussion: Social Acceptance and Efficiency

The survey reflects that a social robot needs to inspire tranquility and comfort, not disturbing people in its surroundings. It has to behave in a predictable way, keeping a safe distance from objects and, especially, from people. It should be aware of social conventions and act accordingly. It seems quite clear that the robot should focus mainly on these factors, and only if they are fulfilled, it can try moving *as a person*.

Trajectories that follow smooth curves are the ones that better suit these requirements. Saw-shaped trajectories can be discarded as they tend to produce exactly the opposite impressions. Straight lines, with 90° turns, could be worthy to perform for the robot, as they keep distance and make the robot appear *naturally as a robot*.

The results of the energy consumption experiment, however, show that these 90° turns are the ones that consume more energy. Smooth curves, on the other hand, are the most efficient option.

The correlation of both experiments allows concluding that trajectories following smooth curves should be selected for a social robot, as they minimize energy consumption and maximize acceptability criteria. It is also clear that a social robot should sacrifice some efficiency, following longer paths, if these paths maintain it as far as possible from humans. While the behavior of a social robot is not to be defined easily, the results of this paper show that, when designing the movement of a social robot, one adequate approach is: (i) firstly, to maximize the distance to people; (ii) moving in smooth trajectories that can be easily predicted, avoiding sharp turns; and (iii) approaching people only when the robot is going to interact with them, or when there is no other available path. In these situations, the robot should be aware of the social context, and tune its behavior to fit it. These results highlight the importance of using path planners that allows efficiently considering different factors, and tuning them online, when dealing with social robotic navigation. Hence, proposals like the ones presented in Reference [23] or [24] are specially interesting to conform the base of these path planners.

## 8. Conclusions and Future Work

The experiments analyzed in this paper reflect that the smoothness factor has a relevant influence on energy consumption and social acceptance; thus, it should be considered when designing the motion of a social robot. In general terms, smoother trajectories will decrease energy consumption and increase acceptability. Abrupt direction changes should always be avoided, and, in the presence of people, the robot should try to keep itself as far as possible from humans, moving in predictable smooth trajectories, while caring about the social context. Being aware of the social context becomes a strong requirement for a social robot if it has to be accepted.

Following the same trajectories that a person would follow may be desirable. However, it is essential to keep in mind that some behaviors that, sometimes, are tolerated in people (like going between two people who are speaking to reach an object in a table) may trigger worse reactions when performed by a robot, at least as long as social robots do not become a really usual presence for us. Hence, social robots should behave like the perfect butler, always available but never disturbing.

This experiment could be improved by taking other considerations on our methods of smoothness calculation. The current paper analyzes the trajectories offline; hence, a manual method was chosen. For our future research, we plan to follow a two-step approach to extend the computation of smoothness:

- Firstly, it will be using offline processing to compute the smoothness factor after the real robot has executed a use case. Once computed, the smoothness value can be correlated with measured energy consumption and perceived social acceptability (this last measure will have to be obtained after the tests, via questionnaires filled by participants in the use case), so that the robot will be able to learn, through supervised offline learning, which paths were more adequate.
- Secondly, the ability to compute smoothness in real-time (while performing) will be incorporated into the robot, so it can use this factor to tune its behavior. For this purpose, an adaptive curvature function algorithm will be used, based on the author’s previous works [26,27] and the approach used in Reference [28].

A future direction would be to study how the different types of wheels can influence energy consumption. Consequently, it would be interesting to perform the same set of experiments with distinct kinds of wheels.

A different approach to future works, it could be to analyze longer paths and more complex scenarios, with more people performing different tasks. Furthermore, it could be stirring for social acceptance to simulate scenarios in which the robot moves using only smooth trajectories, but changing distances from the robot to objects and people, and choosing paths of different lengths. Another important objective is to make surveys more attractive to the participants, so that they remain in the experiment. The surveys could be improved by making them shorter and relying more on free questions: although the information provided by these free questions is more difficult to collect, it has proven to be really useful in understanding the results. Finally, we are planning to perform experiments in real scenarios, in the context of the research projects cited below, in which a social robot will be operating for months in a retirement home.

## Figures and Tables

**Table 1:** Path Planning (PP) algorithm comparison.

Cite	Smoothness	Path Length	Path Safety	Fitness Function	Social Acceptance	Consumption
[5]	X	X	X	X		X
[6]	X	X	X	X		X
[7]	X		X			
[8]	X	X	X	X		X
[9]		X	X	X		
[10]			X		X	
[11]	X					
[12]	X		X			
[13]		X	X	X		
[14]		X	X	X		
[15]		X	X			
[16]		X	X			
[17]		X	X			
[18]	X	X	X			
[19]		X	X			
[20]			X			
[21]	X	X	X	X		X
[22]	X	X	X	X		X
[23]	X	X	X			
[24]	X	X	X			

**Table 2:** Classification of the different paths.

SAW-SHAPED	CURVED	SQUARED	STRAIGHT
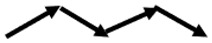	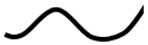	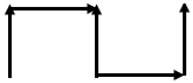	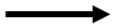

**Table 3:** Battery consumption: No curved paths.

Test	Path	Description	Movements	Consumption	Smoothness
SAW-SHAPED 15°	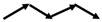	Robot follows a saw-shaped path making 15° with a constant velocity	TURN −15 0.08 MOVE 70 60 Z TURN 30 0.08 MOVE 70 60 Z TURN −30 0.08	5.45 W	150°
SAW-SHAPED 30°	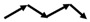	Robot follows a saw-shaped path making 30° with a constant velocity	TURN −30 0.08 MOVE 70 60 Z TURN 60 0.08 MOVE 70 60 Z TURN −60 0.08	5.9 W	120°
SAW-SHAPED 45°	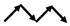	Robot follows a saw-shaped path making 45° with a constant velocity	TURN −45 0.08 MOVE 70 60 Z TURN 90 0.08 MOVE 70 60 Z TURN −90 0.08	6.14 W	90°
SAW-SHAPED 60°	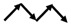	Robot follows a saw-shaped path making 60° with a constant velocity	TURN −60 0.08 MOVE 70 60 Z TURN 120 0.08 MOVE 70 60 Z TURN −120 0.08	6.53 W	60°
SAW-SHAPED 75°	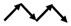	Robot follows a saw-shaped path making 75° with a constant velocity	TURN −75 0.08 MOVE 70 60 Z TURN 150 0.08 MOVE 70 60 Z TURN −150 0.08	8.93 W	30°
SQUARED (90)°	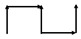	Robot follows a saw-shaped path making 90° with a constant velocity	TURN −90 0.08 MOVE 70 60 Z TURN 180 0.08 MOVE 70 60 Z TURN −180 0.08	10.12 W	90°
STRAIGHT	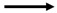	Robot follows a straight path		3.76 W	180°

**Table 4:** Battery consumption: Curved paths.

Test	Path	Description	Movements	Consumption	Smoothness
CURVED WITH V = 0.02	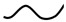	Robot follows a curved path with velocity = 0.02 rad/s and its wheels rotates with an angle = 70º	TURN −45 0.08 ROTATION 70 60 Z 0.02 TURN 45 0.08 ROTATION 70 60 Z 0.02 TURN −90 0.08	5.2 W	34 cm
CURVED WITH V = 0.04	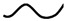	Robot follows a curved path with velocity = 0.04 rad/s and its wheels rotates with an angle = 70º	TURN −45 0.08 ROTATION 70 60 Z 0.04 TURN 45 0.08 ROTATION 70 60 Z 0.04 TURN −90 0.08	4.9 W	36.25 cm
CURVED WITH V = 0.06	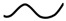	Robot follows a curved path with velocity = 0.06 rad/s and its wheels rotates with an angle = 70°	TURN −45 0.08 ROTATION 70 60 Z 0.06 TURN 45 0.08 ROTATION 70 60 Z 0.06 TURN −90 0.08	4.6 W	38.45 cm
CURVED WITH V = 0.08	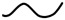	Robot follows a curved path with velocity = 0.08 rad/s and its wheels rotates with an angle = 70°	TURN −45 0.08 ROTATION 70 60 Z 0.08 TURN 45 0.08 ROTATION 70 60 Z 0.08 TURN −90 0.08	4.49 W	45.57 cm
CURVED WITH V = 1.1	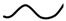	Robot follows a curved path with velocity = 1.1 rad/s and its wheels rotates with a angle = 70°	TURN −45 0.08 ROTATION 55 60 Z 0.1 TURN 45 0.08 ROTATION 55 60 Z 0.1 TURN −90 0.08	1.92 W	58 cm
CURVED WITH V = 1.2	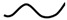	Robot follows a curved path with velocity = 1.2 rad/s and its wheels rotates with an angle = 70°	TURN −45 0.08 ROTATION 45 60 Z 0.12 TURN 45 0.08 ROTATION 45 60 Z 0.12 TURN −90 0.08	1.8 W	74.35 cm

**Table 5:** Scenarios.

Scenarios	Description	Situations	Image
1	The robot goes from the yellow point to the red point. The scene includes one column, one table, and two people standing facing each other. The robot will pass between them.	1.0/1.1/1.2/1.3/1.4	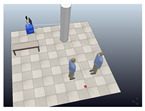
2	The robot goes from the yellow point to the red point. The scene includes one column, one table, and two people standing facing each other. The robot will pass around them.	2.1/2.2/2.3/2.4	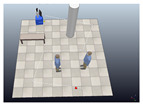
3	The robot goes from the yellow point to the red point. The scene includes two columns and a table with a chair. The robot surrounds all obstacles.	3.1/3.2/3.3/3.4	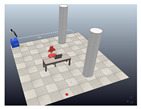
4	The robot goes from the yellow point to the red point. The scene includes two columns, a table with a chair, and a plant. The robot passes between the obstacles.	4.1/4.2/4.3/4.4	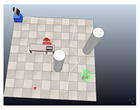
5	The robot goes from the yellow point to the red point. The scene includes two columns and a table with a chair. The robot passes between the obstacles.	5.1/5.2/5.3/5.4	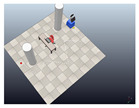

**Table 6:** Situations.

Situation	Description
X.0	The robot moves following a straight line.
X.1	The robot follows a path in which only 90° turns are allowed.
X.2	The robot follows a saw-shaped path.
X.3	The robot follows a path with smooth curves.
X.4	The robot follows a path with very smooth curves.

**Table 7:** Smoothness simulated scenarios.

Situations	Smoothness Average	Smoothness Standard Deviation
1.0	180°	0
1.1	90°	0
1.2	75°	10.80
1.3	19.81 cm	0.13
1.4	50.27 cm	0.35
2.1	90°	0
2.2	73.75°	12.93
2.3	10.2 cm	0.03
2.4	48.59 cm	0.16
3.1	90°	0
3.2	55°	4.08
3.3	86.36 cm	0.17
3.4	137.63 cm	1.16
4.1	90°	0
4.2	73°	4.08
4.3	12.63 cm	0.07
4.4	36.37 cm	0.12
5.1	90°	0
5.2	61°	7.35
5.3	12.32 cm	0.07
5.4	30.12 cm	0.16

**Table 8:** Links for each survey.

Survey	Link
Case 01	https://forms.gle/R64TjJQDXZ7WEhdQ7
Case 02	https://forms.gle/x9qBvGHabaHAU17u6
Case 03	https://forms.gle/2D8RdLWpmFXVYexx5
Case 04	https://forms.gle/Mq1avDdr7QVunP8C7
Case 05	https://forms.gle/hEeBeTvYPKbtk6UC8

**Table 9:** Questionnaire items for each situation.

Question	Description
Q1	The robot perceives/takes people into account.
Q2	The robot’s actions are clear to people.
Q3	Robot navigation is appropriate from the point of view of our social conventions.
Q4	The movement of the robot is similar to a person in the same situation.
Q5	The robot perceives/understands the social context of the situation described in the video.
Q6	If you were one of the two people in the room, would you feel threatened by the robot?
Q7	Is the robot’s movement unpredictable?
Q8	If you were one of the two people in the room, would you mind if the robot was navigating the room autonomously?
Q9	Would it distract you from your usual tasks?

**Table 10:** Open questions in the closing section of the survey.

Open Question	Description
OQ1	Would you have preferred some other movement of the robot that you have not seen in the videos? Describe the displacement.
OQ2	Which of the 5 trajectories did you find more natural and less invasive? Justify your answer.

**Table 11:** Demographic data of the participants.

Gender		Age				
**Male**	**Female**	**20 to 30**	**30 to 40**	**40 to 50**	**50 to 60**	**60 to 70**
67.22%	32.78%	46.05%	20.61%	25.81%	6.40%	1.13%

**Table 12:** Experience of the participants.

Experience Using Technology				Experience with Robots		
**Mobile/Tablet**	**Computer**	**Administrator**	**Programmer**	**No**	**Yes**	**Few**
88.32%	84.18%	37.02%	67.36%	56.54%	20.94%	22.54%

**Table 13:** Results of the survey for the Likert Scale questions (Q1–Q7).

Situation	Q1	Q2	Q3	Q4	Q5	Q6	Q7
1.0	2.50 ± 1.39	4.02 ± 1.24	2.89 ± 1.52	3.11 ± 1.62	2.11 ± 1.24	2.85 ± 1.20	1.85 ± 1.27
1.1	3.11 ± 1.43	3.15 ± 1.33	2.65 ± 1.40	1.87 ± 1.19	2.28 ± 1.33	2.24 ± 1.15	2.70 ± 1.14
1.2	2.91 ± 1.40	2.17 ± 1.15	1.87 ± 1.10	1.41 ± 0.77	2.13 ± 1.26	3.09 ± 1.43	3.52 ± 1.23
1.3	3.20 ± 1.38	2.76 ± 1.29	2.26 ± 1.21	2.04 ±1.00	2.24 ± 1.22	2.65 ± 1.25	3.20 ± 1.19
1.4	3.28 ± 1.28	3.11 ± 1.17	2.87 ±1.23	2.52 ± 1.12	2.35 ± 1.20	2.30 ± 1.16	2.65 ± 1.11
2.1	4.50± 0.58	3.33 ± 1.31	3.92 ± 1.32	2.58 ± 1.38	3.54 ± 1.32	1.96 ± 1.27	2.67 ± 1.31
2.2	4.13 ± 0.73	2.54± 1.15	3.29 ± 1.31	1.79 ± 0.91	3.38 ± 1.35	2.29± 1.17	3.17 ± 1.34
2.3	4.29 ± 0.79	3.63 ± 1.07	4.08 ± 1.11	2.96 ± 1.21	3.63 ± 1.28	2.21 ± 1.32	2.33 ± 1.07
2.4	4.29 ± 0.93	3.92 ± 1.19	4.17 ± 0.80	3.75 ± 1.09	3.67 ±1.28	1.79 ± 1.00	1.96 ± 1.02
3.1	3.95 ± 1.17	4.19 ± 0.96	4.38 ± 0.95	0.79 ± 1.08	3.48 ± 1.22	1.76 ± 0.97	1.90 ± 0.97
3.2	3.57 v 0.90	2.33 ± 1.13	2.76 ± 1.60	1.57 ± 0.79	2.24 ± 1.34	2.95 ± 1.36	3.48 ± 1.18
3.3	4.05 ± 1.04	4.24 ± 0.87	4.38 ± 0.65	3.62 ± 1.21	3.62 ± 1.25	1.48 ± 0.73	1.57 ± 0.73
3.4	4.14 ± 0.99	4.43 ± 0.73	4.33 ± 0.71	3.48 ± 1.10	3.76 ± 1.19	1.67 ± 1.04	1.67± 0.84
4.1	3.87 ± 1.31	2.93 ± 1.12	2.73 ± 1.44	1.47 ± 0.62	2.87 ± 1.02	2.67 ± 1.53	2.93 ± 1.34
4.2	3.47 ± 1.09	2.53 ± 1.20	2.47 ± 1.31	1.60 ± 0.72	2.73 ± 1.00	2.67 ± 1.40	3.27 ± 1.44
4.3	3.53 ± 1.31	2.93 ± 1.44	3.13 ± 1.41	2.60 ± 1.31	2.93 ± 1.12	2.33 ± 1.25	2.60 ± 1.45
4.4	3.60 ±1.25	3.53 ± 1.31	3.87 ± 1.15	3.40 ± 1.08	3.40 ± 1.25	1.80± 0.91	2.00 ± 0.89
5.1	3.00 ± 1.65	2.07 ± 1.28	1.93 ± 1.28	1.50 ± 0.73	1.79± 1.01	3.36 ± 1.59	4.07 ± 1.33
5.2	2.71 ±1.44	2.07 ± 1.10	1.93 ± 1.10	1.36 ± 0.61	2.14 ± 0.99	3.21 ± 1.66	3.79 ± 1.26
5.3	3.29 ± 1.22	3.00 ± 1.00	2.64 ± 0.89	2.36 ± 0.81	2.79 ± 0.94	2.57 ± 1.45	2.64 ± 1.44
5.4	3.57 ± 1.29	3.29± 1.22	2.93 ± 1.22	2.71 ±0.96	3.00± 1.00	2.57 ± 1.40	2.57± 1.35

**Table 14:** Results of the survey (percentage of agreement).

Situation	Q8	Q9
1.0	21.7	37
1.1	28.3	47.8
1.2	43.5	56.5
1.3	37	52.2
1.4	28.3	43.5
2.1	12.5	25
2.2	29.2	33.3
2.3	20.8	25
2.4	16.7	16.7
3.1	0	19
3.2	42.9	61.9
3.3	4.8	14.3
3.4	9.5	9.5
4.1	40	53.3
4.2	60	46.7
4.3	40	40
4.4	26.7	26.7
5.1	50	64.3
5.2	57.2	57.1
5.3	35.7	42.9
5.4	42.9	42.9

## Abbreviations

The following abbreviations are used in this manuscript:

AI	Artificial Intelligence
ACS712	Sensor to measure the current
AGV	Autonomous Ground Vehicles
API	Application Programming Interface
EC	Evolutionary Computing
FMM	Fast Marching Method
FZ0430	Sensor to measure the voltage
GA	Genetic Algorithm
HC-05	Bluetooth module
MOO	Multi-Objective Optimization
MOVNS	Multi-Objective Variable Neighborhood Search
MOPSO	Multi-Objective Particle Swarm Optimization
MQTT	Message Queuing Telemetry Transport
NSGA	Non-Dominated Sorting Genetic Algorithms
NUC	Next Unit of Computing
PC	Personal Computer
PO	Pareto
PP	Path Planning
PSM	Path Segment Method
ROS	Robot Operating System
RS-485	Electrical characteristics of drivers and receivers for use in serial communications systems
UAV	Unmanned Aerial Vehicles
WiFi	Wireless Fidelity
